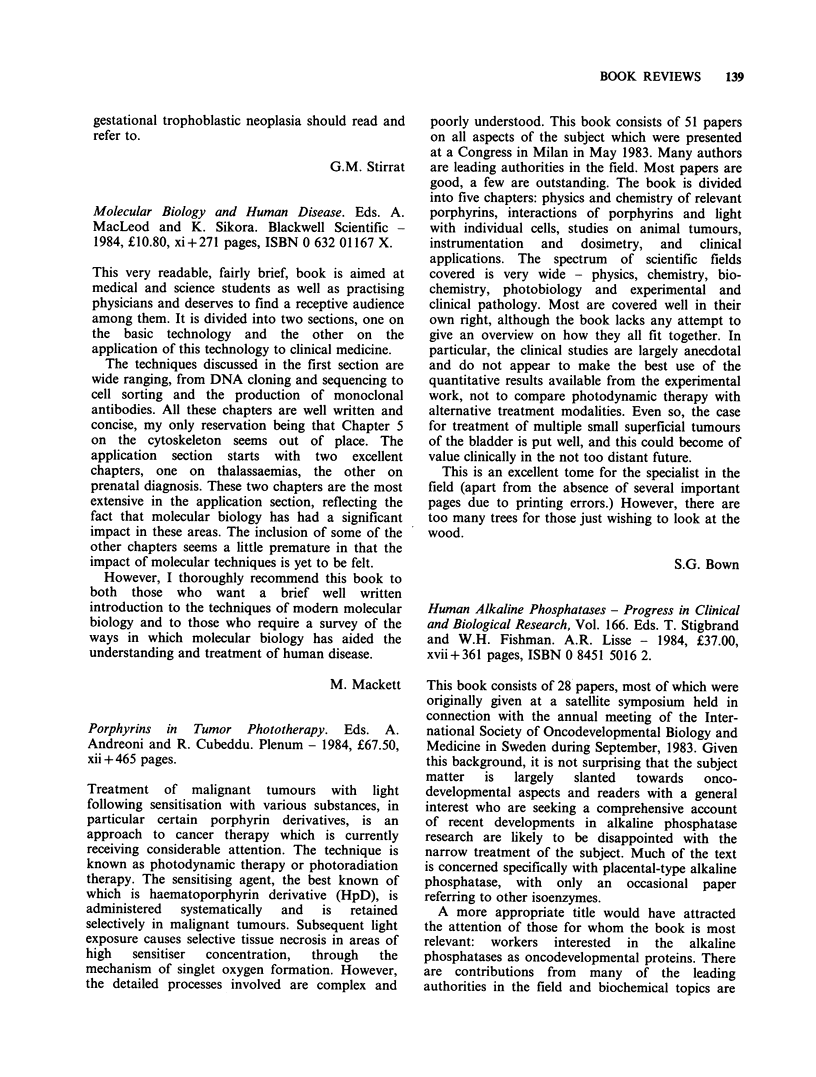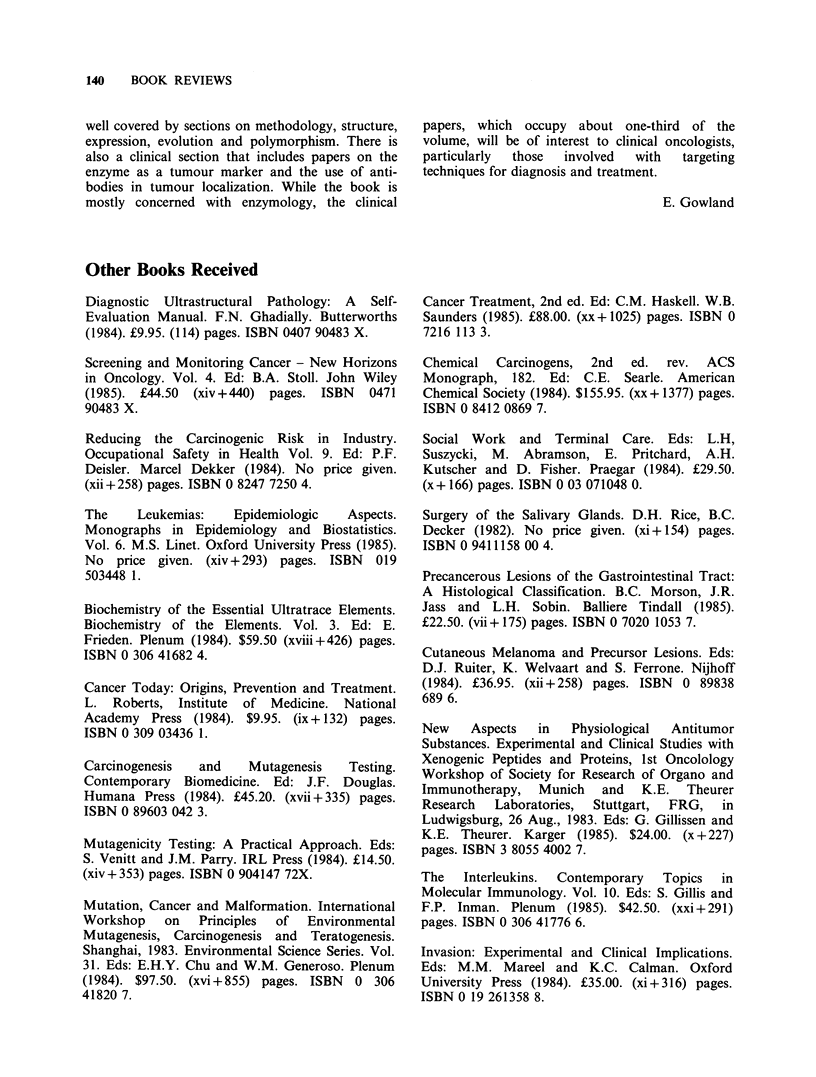# Human Alkaline Phosphatases - Progress in Clinical and Biological Research

**Published:** 1985-07

**Authors:** E. Gowland


*Human Alkaline Phosphatases – Progress in Clinical and Biological Research,* Vol. 166. Eds. T. Stigbrand and W.H. Fishman. A.R. Lisse – 1984, £37.00, xvii+361 pages, ISBN 0 8451 5016 2.

This book consists of 28 papers, most of which were originally given at a satellite symposium held in connection with the annual meeting of the International Society of Oncodevelopmental Biology and Medicine in Sweden during September, 1983. Given this background, it is not surprising that the subject matter is largely slanted towards oncodevelopmental aspects and readers with a general interest who are seeking a comprehensive account of recent developments in alkaline phosphatase research are likely to be disappointed with the narrow treatment of the subject. Much of the text is concerned specifically with placental-type alkaline phosphatase, with only an occasional paper referring to other isoenzymes.

A more appropriate title would have attracted the attention of those for whom the book is most relevant: workers interested in the alkaline phosphatases as oncodevelopmental proteins. There are contributions from many of the leading authorities in the field and biochemical topics are well covered by sections on methodology, structure, expression, evolution and polymorphism. There is also a clinical section that includes papers on the enzyme as a tumour marker and the use of antibodies in tumour localization. While the book is mostly concerned with enzymology, the clinical papers, which occupy about one-third of the volume, will be of interest to clinical oncologists, particularly those involved with targeting techniques for diagnosis and treatment.

E. Gowland